# Draft Genome Sequence of Listeria monocytogenes Clonal Complex 1 Strain SNU3 from South Korea

**DOI:** 10.1128/mra.01226-22

**Published:** 2023-01-25

**Authors:** Sangmi Lee, Sangryeol Ryu

**Affiliations:** a Department of Food and Nutrition, Chungbuk National University, Cheongju, Chungbuk, South Korea; b Department of Food and Animal Biotechnology, Seoul National University, Seoul, South Korea; University of Maryland School of Medicine

## Abstract

Currently, only limited knowledge is available about the genetic diversity of the hypervirulent clonal complex 1 (CC1) of Listeria monocytogenes from Asia. In this study, we report the draft genome sequence of an L. monocytogenes CC1 strain (SNU3) from Seoul, South Korea.

## ANNOUNCEMENT

Listeria monocytogenes is a Gram-positive foodborne pathogen that causes listeriosis, a disease that manifests with severe, potentially lethal symptoms (1–4). The L. monocytogenes population includes several hypervirulent clones that are often disproportionately responsible for clinical cases, considering the frequency among food isolates (5). Clonal complex 1 (CC1) is one such hypervirulent clone that has caused multiple outbreaks in North America and Europe and has been identified worldwide (5–8). While the genetic diversity and evolutionary history of this large clonal group were extensively investigated by Moura et al., most strains included in their study originated from Europe and North America, leaving a large gap in our understanding of the genetic diversity of CC1 strains from other geographical regions, such as Asia (8). In this study, we report the draft genome sequence of a CC1 strain (SNU3) that was isolated in Seoul, South Korea (9).

SNU3 was isolated in 2004 from a chicken using a protocol recommended by the U.S. Food and Drug Administration (9). Genomic DNA was extracted using the NucleoSpin microbial DNA kit (Macherey-Nagel, Duren, Germany) from an overnight culture grown at 37°C on a brain heart infusion (Becton, Dickinson and Co., Sparks, MD, USA) agar plate, and a paired-end library was prepared using the TruSeq Nano DNA library prep kit (Illumina, San Diego, CA, USA). Genome sequencing using the MiSeq (Illumina) platform (2 × 300 bp) generated 1,641,989 pairs of reads, with a total length of 840.1 Mbp, whose quality was checked using FastQC v0.11.9 (10). Trimmomatic v0.38 (11) was employed with modified parameters (LEADING:10 TRAILING:10 SLIDINGWINDOW:4:20 MINLEN:200) to trim adaptors and eliminate reads with a quality score below 20, and BWA v0.7.17 (12) was used to remove sequences from the PhiX Control v3 library (Illumina), which were included as the sequencing control. The resulting 899,973 pairs of reads were *de novo* assembled using SPAdes v3.13.0 (13) into 13 contigs with a total length of 2,986,681 bp, a coverage of 154.36×, an *N*_50_ value of 405,169 bp, and a GC content of 37.83%. Multilocus sequence typing (MLST) analysis using the *Listeria* MLST database maintained by Institut Pasteur (https://bigsdb.pasteur.fr/listeria/) revealed that SNU3 belongs to sequence type 1 and CC1. Genome annotation was performed using the NCBI Prokaryotic Genome Annotation Pipeline v6.3 (14), which identified 2,935 protein-coding sequences, 26 pseudogenes, 6 rRNAs, and 54 tRNAs in the annotated draft genome. All software was run with default parameters unless otherwise stated. The genome sequence data in this announcement will contribute to unraveling the full diversity of CC1 at the global level, which could be instrumental for effectively tracking down and controlling L. monocytogenes outbreaks in the era of the international food chain.

### Data availability.

The draft genome sequence of L. monocytogenes strain SNU3 was deposited at GenBank under the accession no. JAPFBZ000000000 (BioProject accession no. PRJNA898764 and BioSample accession no. SAMN31621833). The version described in this paper is the first version. The raw sequencing reads have been deposited in the SRA under the accession no. SRR22356289.
